# Chapin A. Harris

**Published:** 1895-06

**Authors:** Samuel J. Cockerille


THE
AMERICAN JOURNAL
—OF—
DENTAL SCIENCE.

VOL. XXIX. BALTIMORE, JUNE, 1895. NO. 2.

ARTICLE I.

### CHAPIN A. HARRIS, M. D., D. D. S.

BY. SAMUEL J. COCKERILLE.

*Mr. President and Brothers of Maryland Dental Society:—*

I hold in my hand an address made to the Virginia Dental Society. With your consent I will now read it. It will require only a few minutes. It is an effort to get justice to the memory of the noble Chapin A. Harris, the father of our glorious profession. I will then endeavor to show why every dentist in Maryland should lay hold of the good work and push it to a grand ending.

You are greatly indebted to the noble men of your own state. While rememberıng and honoring Harris and his work do not forget that Maryland gave to the Profession the most learned man and greatest teacher she ever had—Philip H. Austen. You should not allow these names and those of Hayden, Handy, Bond and Piggott, to pass into oblivion.

While ingratitude is the basest of sins, gratitude is the sweet smelling virtue that makes its home in the manly and pure heart. The works and valuable services of Harris forgotten? The great virtue, gratitude dead in your minds, hearts and souls? No, not dead, but sleeping; and we are here to day to rouse you from your slumber and press you into action, and remind you of the duty of performance. Harris' name should be dear to every dentist, and especially so to this Society, and to each and every dentist, and of this State. His virtues and merits demand a monument of this State. It is a most laudable undertaking, and surely will receive approval of the mind, heart and soul of every member of our glorious Profession for the perpetuation of an honorable fame. Sixteen thousand dentists in this land of free institutions allow their father's name to pass into oblivion unhonored and unsung? Will not our brothers of the great city of Baltimore, and State of Maryland make it a labor of love, as it is their duty to erect a monument to this good, great and noble man? The marvelous acts of self-sacrifice to which his love of dentistry and brotherly love for mankind gave birth, should be blazoned before the eyes of the world, for emulation by those who are to enjoy the benefits of dentistry after we have passed over the river.

We know you have an admiration and reverence for his great character; you could not have less for the father of your profession; then put forth your greatest and noblest efforts to honor his fame and name. The responsibilities of life are great. With truth, honesty and determination he was always striving for the right, and at the same time striving to induce the less fortunate to abandon the errors into which they have fallen. He was a man of character who looked at the heart, and not at the arm; he weighed the justice of his cause, and not its outward strength. The question settled his path was clear. He went forward boldly, were it even against the entire profession, and looking for reward only in the conscious fact of duty done, and the good the human family would reap. He did not allow himself to be driven from the right.

Dollars are not the only thing in the world. In the right, we are never alone. The Great Master is always with those in the right; and therefore they will always be on the strong side. When we are battling truth against error, we should have no internal uneasiness. If we are fortified with steadiness of purpose and energy we are sure to stand. His life was a grand success, because he was always fortified with steadiness of purpose and energy. He knew the muscular power, the nervous strain and great skill required in doing full duty to dentistry; and that the true man would not allow himself to be tempted and drawn into the wrong. He taught us to labor for truth, not with passion and hypocrisy, but with integrity of purpose, with determination to maintain what is true although contrary to dollars, numbers and any system. The enthusiasm which with many is fitful and spasmodic, was with him the current of his life; solemn and deep as the tide of his life. Chapin A. Harris was unselfish and ambitious, but his ambition was manifested by industry and energy to gain all knowledge possible and was at all times ready to distribute and willing to communicate.

We regard the establishment of the Baltimore Dental College as the epoch in the history of our profession, and we admire the life and character of the man to whom we are most indebted for the great advantages dentistry has conferred upon us, and the invaluable blessings which have flowed from his matchless work. We know you appreciate it, and you should feel there is a duty resting upon you and show respect and reverence for his noble efforts, so that his work may be handed down—the admiration of future generations. He spent his unselfish and ambitious life in the service of dentistry, and you should rise in your might and discharge a debt of gratitude to his memory, and erect a *monument* that will be a lasting evidence of your thanks and your love to him who gave to us our glorious profession. Harris toiled for her advancement from night till morn; from morn till dewy eve; the

Baltimore Dental College and the principles and practice of dentistry, are time-honored and lasting monuments of the essence of his wisdom. The richest heritage he could have bestowed. You and I profited by these advantages. What would we have been without them?

Duty neglected!—a fearful thought!—Harris—the inspiration of your first love. What was the birth, purpose and original first love of dentistry? It was born of a consuming desire to do good to our fellow-man. Harris saw the framework of a great profession—dead laymen ministered it its altars—they wandered like sheep without a shepherd. It was out of this necessity that your profession came. Harris died in poverty, but in glory; for he had laid the foundation of dentistry which when properly appreciated will become the wonder of the world. Take not your eyes off its birth purposes. Be true to that birthright. When you fail to devote yourself to the work Harris set apart for you, your occupation will be gone. There will be no place for dentistry among honorable professions. Remember your birthright, and be true to your mission.

To our younger brothers we say never forget it is more honorable to lay off the harness, than to put it on. It is a noble and great thing to cover the blemishes and excuse the failings and short-comings of a professional brother and friend: to draw a curtain over his stains, and to display his perfections; to bury his weaknesses in silence, and to proclaim his virtues and his good qualities from the house-top. Imitate as far as possible the love of Heaven. Love hides a multitude of sins. Let your thirst after knowledge be equaled only by your desire to communicate what you have learnt. Make your influence far-reaching and extensive, and who can estimate your good to the profession and to mankind? Make your books your constant companions. If you wish to achieve a bright destiny in your profession; you must work with your books as faithfully as it is possible to labor with your instruments. Will you by pursuing such a course, fail? We trust not, although you will have to encounter black and lowering clouds if not storms—let troubles come as they may, be earnest and determined; and success is sure to follow..

Walk in conformity to art, and science, and good example; not only injuring no man, but doing good to all. Never hesitate between conscience and interest, but with courage and energy attack errors and mistakes.

Trust not in man, but ever remember that God strives to lead us right, often unfelt when helping the most; unseen, when nearest of all.

MR. PRESIDENT, we regard it as the duty of this Society to feel they have an exalted mission to fill, and it is their bounden right to do all that is possible to honor the noble man who raised dentistry from a low and ignominious standing, and gave her the rank of a glorious profession.

We have attempted to render to you no new version of his life; we do not forget that we are standing in a City and State where every dentist holds his memory sacred, and a rehearsal of a story long ago learned and treasured in your hearts would only prove a tax upon your patience—carrying coals to Newcastle.

In your efforts draw together as one man, with a single heart and common purpose, and you are sure of success.

Chapin A. Harris was an inspiring example of fidelity to truth and duty; a landmark and Polar star, for the guidance of all who love their fellow man; and for all who worship virtue and honor science.

---

ARTICLE II.

